# CCL12 induces trabecular bone loss by stimulating RANKL production in BMSCs during acute lung injury

**DOI:** 10.1038/s12276-023-00970-w

**Published:** 2023-04-03

**Authors:** Chao Ma, Juan Gao, Jun Liang, Feizhen Wang, Long Xu, Jinhui Bu, Bo He, Guangpu Liu, Ru Niu, Guangwang Liu

**Affiliations:** 1 Department of Orthopedic Surgery, Xuzhou Central Hospital, Xuzhou Clinical School of Xuzhou Medical University, Xuzhou Central Hospital Affiliated to Nanjing University of Chinese Medicine, The Xuzhou School of Clinical Medicine of Nanjing Medical University, Xuzhou Central Hospital Affiliated to Medical School of Southeast University, 199 Jiefang South Road, Xuzhou, 221009 China; 2 Department of Gynecology and Obstetrics, Xuzhou Central Hospital, Xuzhou Clinical School of Xuzhou Medical University, Xuzhou Central Hospital Affiliated to Nanjing University of Chinese Medicine, The Xuzhou School of Clinical Medicine of Nanjing Medical University, Xuzhou Central Hospital Affiliated to Medical School of Southeast University, 199 Jiefang South Road, Xuzhou, 221009 China; 3 Department of Endocrinology, Xuzhou Central Hospital, Xuzhou Clinical School of Xuzhou Medical University, Xuzhou Central Hospital Affiliated to Nanjing University of Chinese Medicine, The Xuzhou School of Clinical Medicine of Nanjing Medical University, Xuzhou Central Hospital Affiliated to Medical School of Southeast University, 199 Jiefang South Road, Xuzhou, 221009 China

**Keywords:** Acute inflammation, Osteoporosis, Mechanisms of disease, Mesenchymal stem cells

## Abstract

In the last three years, the capacity of health care systems and the public health policies of governments worldwide were challenged by the spread of SARS-CoV-2. Mortality due to SARS-CoV-2 mainly resulted from the development of acute lung injury (ALI)/acute respiratory distress syndrome (ARDS). Moreover, millions of people who survived ALI/ARDS in SARS-CoV-2 infection suffer from multiple lung inflammation-induced complications that lead to disability and even death. The lung-bone axis refers to the relationship between lung inflammatory diseases (COPD, asthma, and cystic fibrosis) and bone diseases, including osteopenia/osteoporosis. Compared to chronic lung diseases, the influence of ALI on the skeleton has not been investigated until now. Therefore, we investigated the effect of ALI on bone phenotypes in mice to elucidate the underlying mechanisms. In vivo bone resorption enhancement and trabecular bone loss were observed in LPS-induced ALI mice. Moreover, chemokine (C-C motif) ligand 12 (CCL12) accumulated in the serum and bone marrow. In vivo global ablation of CCL12 or conditional ablation of CCR2 in bone marrow stromal cells (BMSCs) inhibited bone resorption and abrogated trabecular bone loss in ALI mice. Furthermore, we provided evidence that CCL12 promoted bone resorption by stimulating RANKL production in BMSCs, and the CCR2/Jak2/STAT4 axis played an essential role in this process. Our study provides information regarding the pathogenesis of ALI and lays the groundwork for future research to identify new targets to treat lung inflammation-induced bone loss.

## Osteoporosis: Interrupting inflammation-induced bone loss

Lung inflammation stimulates the production of signaling molecules that cause stem cells in the marrow to drive accelerated bone loss. Several studies have linked respiratory inflammation in disorders like asthma and chronic obstructive pulmonary disease to osteoporosis, but the pathways linking lung and bone remain poorly understood. Guangwang Liu at Xuzhou Central Hospital, China, and colleagues have investigated this connection by assessing skeletal damage in mice after lung injury due to inhalation of a bacterial toxin. Within weeks, the researchers observed high levels of a protein called CCL12 in the bone marrow. This protein acts on stem cells in the marrow and stimulates bone degeneration. This bone loss could be prevented by either blocking CCL12 activity or knocking out its coding gene, revealing a potential opportunity to protect skeletal integrity in patients with pulmonary disorders.

## Introduction

In the last three years, the capacity of health care systems and the public health policies of governments worldwide were challenged by the spread of severe acute respiratory syndrome coronavirus 2 (SARS-CoV-2)^1^. Mortality due to SARS-CoV-2 infection resulted mainly from the development of inflammation-induced acute lung injury (ALI) and its most severe manifestation, acute respiratory distress syndrome (ARDS)^2^. Despite studies and advances regarding SARS-CoV-2, such as vaccines, which have clearly been important in controlling infection rates^3^, the pathophysiological mechanisms that trigger ALI/ARDS are still not fully understood. Moreover, hundreds of millions of people who survived ALI/ARDS in SARS-CoV-2 infection suffer from multiple lung inflammation-induced complications that lead to disability and even death^4^. Understanding the mechanisms underlying these complications is vital to finding effective ways to protect against illness.

Given its central role in the circulatory system, the lungs can communicate with other organs through an extraordinary variety of soluble factors through blood flow. The lung-bone axis refers to the relationship between lung inflammatory diseases and bone diseases, including osteopenia/osteoporosis. Osteoporosis and fracture are recognized as systemic features of several chronic inflammatory lung diseases, including chronic obstructive pulmonary disease (COPD), asthma, and cystic fibrosis, and can occur after lung transplantation^5–9^. In COPD and asthma, osteoporosis can still occur even if proven osteoporosis risk factors are absent, such as age, nutrition, medications, body mass index, cigarette smoking, and sex^8,9^. This finding suggests that lung inflammation is an independent and important risk factor for bone loss. Compared to chronic lung diseases, the effects of acute lung injury on the skeleton have not been investigated. In the current study, we revealed trabecular bone loss in mice with LPS-induced acute lung injury, and chemokine (C-C motif) ligand 12 (CCL12) played an important role in this process. Therefore, the aim of this study was to investigate the underlying molecular mechanisms.

CCL12, which is also known as monocyte chemotactic protein 5 (MCP-5), is mainly produced by astrocytes and Mφs and can attract lymphocytes, monocytes, and eosinophils^10,11^. CCL12 exerts biological effect by binding to its receptor CCR2, which is a member of the G protein-coupled receptor (GPCR) family and is expressed on the cell membrane of memory T cells, NK cells, Mφs, dendritic cells, monocytes, and basophils^12,13^. Few studies have reported the role of CCL12 in the skeleton. A previous study reported that CCL12 regulated joint formation and limb ossification through CCR2 during development^14^. The same research group further revealed the potential efficacy of antagonizing CCR2 during the early stages of OA to slow the progression of postinjury OA and improve pain symptoms^15^. Another study provided evidence that CCL12 in the perichondrium was downregulated by IKKβ and involved in the endochondral ossification of the growth plate^16^. However, there have been no reports concerning the role of CCL12 in osteoporosis. In the current study, we revealed for the first time that CCL12 accumulated in the bone marrow of ALI mice and promoted trabecular bone loss by stimulating RANKL production in bone marrow stromal cells (BMSCs). In vivo global ablation of CCL12 or conditional ablation of CCR2 in BMSCs abrogated trabecular bone loss in ALI mice. The Jak2/STAT4 axis plays an important role in CCL12-mediated activation of RANKL expression in BMSCs. Our study provides evidence regarding the pathogenesis of ALI and lays the groundwork for future research to identify new targets to treat lung inflammation-induced trabecular bone loss.

## Materials and methods

### Animals

The animal protocol was reviewed and approved by the Animal Ethics Committee of Xuzhou Central Hospital. All animal experiments complied with the ARRIVE guidelines and were carried out in accordance with the National Research Council’s Guide for the Care and Use of Laboratory Animals. Male wild-type C57BL/6 mice were obtained from SIPPR-BK Laboratory Animal Co. Ltd. Prx1-Cre transgenic mice and CCL12^−/−^ mice were obtained from the Jackson Laboratory (Bar Harbor, Maine, USA). CCR2^fl/fl^ transgenic mice were established by inserting the loxP sites flanking exon 3 of CCR2 into the mouse genome. CCR2^Prx1^ conditional knockout mice were generated by serial breeding of CCR2^fl/fl^ mice with Prx1-Cre transgenic mice under the control of the Prx1 promoter. CCR2 was deleted from BMSCs in CCR2^Prx1^ mice. All mice were housed under SPF conditions with a 12 h light/dark cycle and were given free access to food and water.

Before the samples were harvested, the animals were euthanized by isoflurane inhalation anesthesia followed by cervical dislocation. Femurs were collected for micro-CT scans, bone marrow cell preparation and bone histomorphometry analysis. Peripheral blood was collected by intracardiac puncture for antibody array analysis.

The administration of in vivo neutralizing antibodies against chemokines is described in the Supplementary data.

### LPS-induced ALI

LPS-induced ALI was established as previously described^17^. Briefly, male mice (8–10 weeks old) were intratracheally (i.t.) instilled with 5 mg/kg LPS (Sigma-Aldrich, St Louis, MO) under anesthesia with 50 mg/kg pentobarbital. Control mice were i.t. instilled with saline. The day when LPS was administered was defined as “Day 0”. Peripheral blood, lung tissue, and bronchoalveolar lavage fluid (BALF) were harvested on Days 0, 2, 8, and 14. The total protein concentration in BALF was measured using a BCA kit (Pierce, Rockford, IL, USA). The concentrations of TNF-α and IL-6 in BALF were measured with ELISA kits (R&D Systems, Minneapolis, MN, USA). The measurement of myeloperoxidase (MPO) activity in lung tissue is described in the Supplementary data. Femurs were collected on Day 14.

### Lung histology

Lung histology was performed as previously described^18^. Briefly, the lungs were fixed with 4% paraformaldehyde and embedded in paraffin. Section (3 μm thickness) were stained with hematoxylin and eosin (H&E). Each slide was reviewed and semiquantitatively assessed to determine the degree of lung inflammation according to a previously published scoring system by two professional researchers who were blinded to the treatment conditions^19^. The scoring system was a Likert scale (0–3), and higher values represented increased histopathologic inflammation.

### Micro-CT

Micro-CT was performed as previously described^20^. Femurs were collected and preserved in 70% ethanol. The bones were scanned using a micro-CT instrument (μCT-80, Scanco Medical AG, Bassersdorf, Switzerland). Standard nomenclature and guidelines were followed, as recommended by the American Society for Bone and Mineral Research^21^. The bones were scanned at an energy level of 55 kVp, an intensity of 145 μA, and a fixed threshold of 220. Trabecular and cortical regions of the femur were analyzed 0.35 mm and 4.25 mm from the growth plate of the distal femur, respectively. For both regions, 1.5 mm femur sections were individually analyzed at a resolution of 6 μm. 3-D images were constructed. The main parameters of trabecular bone are BV/TV (bone volume/total volume), Tb.N (trabecular bone number), and Tb.Sp (trabecular bone space). The main parameters of cortical bone were the Ct.ar/Tt.ar (cortical bone area/total area) and Ct.Th (cortical bone thickness).

### Bone histomorphometry analysis

Femurs were subjected to histomorphometry analyses. All analyses were performed according to the criteria of the American Society for Bone and Mineral Research^22^. Briefly, femurs were decalcified, embedded in paraffin, and sectioned at a thickness of 5 μm onto slides. H&E staining was used to measure bone formation parameters, including the Ob.S/BS and Ob.N/B.Pm. Tartrate-resistant acid phosphatase (TRAP) staining was used to measure bone resorption parameters, including the Oc.S/BS and Oc.N/B.Pm.

### Antibody array

As previously described^23^, an absolute quantitative sandwich-based antibody array (RayBio®) was used to examine 11 chemokines. The detection antibodies were biotin-labeled and mixed as a cocktail for later use. After blocking, the mixtures were incubated with peripheral serum samples. After being washed, a cocktail of biotinylated detection antibodies was added to the arrays. Following incubation and washing, the array slides were incubated with a streptavidin-conjugated fluor (HiLyte Fluor™ 532, from Anaspec, Fremont, CA, USA). Fluorescent signals were visualized using a laser-based scanner system (GenePix 4200 A, Molecular Dynamics, Sunnyvale, CA, USA).

### Immunohistochemistry

Femurs were fixed in 4% paraformaldehyde, decalcified, and embedded in paraffin. Serial sections were cut at a thickness of 5 μm. The slides were incubated with primary antibodies against CCL12 (Biorbyt, Cambridge, United Kingdom), RANKL (Thermo Fisher Scientific, Ann Arbor, MI, USA), and OPG (Thermo Fisher Scientific) overnight at 4 °C. For immunohistochemical staining, a horseradish peroxidase-streptavidin detection system (Dako) was used to detect immunoactivity. Isotype IgG was used as a negative control. Five slides in each sample were selected to determine the integrated optical density (IOD) under a light microscope using ImageJ software.

### Quantitative RT‒PCR (qRT‒PCR)

Total RNA was prepared using an RNeasy Mini Kit (Qiagen, Valencia, CA, USA). Single-stranded cDNA was reverse transcribed from 1 μg of total RNA using oligo-dT primers. Quantitative PCR was performed on an ABI Prism 7500 system (Applied BioSystems, Foster City, CA, USA) and SYBR Green PCR Master Mix (Takara Bio Inc., Otsu, Japan). The cycling conditions were as follows: 94 °C for 5 s; 60 °C for 34 s; and 72 °C for 40 s for 40 cycles. α-Tubulin was used as an internal control. The primer sequences are listed in Supplementary Table 1.

### BMSC isolation and in vitro cell culture

Femur bone marrow was suspended in PBS and passed through a filter. The filtered bone marrow cells were suspended in PBS containing 2% FBS, incubated with a murine progenitor enrichment cocktail (Stem Cell Technologies, Vancouver, BC, Canada) on ice for 30 min, washed, and then seeded onto culture plates at a density of 1 × 10^5^ cells/cm^2^ in α-MEM containing 10% FBS, 100 units/mL penicillin (Gibco BRL, Rockville, MD, USA) and 100 μg/mL streptomycin (Gibco). The media was changed after 3 days, and adherent cells were cultured with twice weekly media changes. BMSCs at passages <3 were used in the current study.

BMSCs were treated in vitro with 50 and 100 ng/ml recombinant mouse CCL12 (R&D Systems, Minneapolis, MN).

### Chromatin immunoprecipitation (ChIP)

As described previously^20^, cells were fixed with 1% formaldehyde for 10 min at 37 °C, and nuclei were prepared. The crude nuclei were subjected to sonication to produce chromatin fragments approximately 500 bp in length. The antibodies included anti-STAT3 (Cell Signaling Technology, Danvers, MA, USA) and anti-STAT4 (Cell Signaling Technology). Isotype IgG (Cell Signaling Technology) was used as a negative control. Primary antibodies (2–5 μg) and the samples were incubated overnight at 4 °C with gentle shaking. The primer sequences for ChIP‒qPCR are listed in Supplementary Table 2. ChIP regions within the RANKL promoter are displayed in Supplementary Fig. 9.

### Western blot analysis

As described previously^20^, total protein was prepared by lysing the cells on ice for 30 min in a buffer (50 mM Tris-HCl, 150 mM NaCl, 1% Nonidet P-40, and 0.1% SDS supplemented with protease inhibitors). The proteins were separated by SDS‒PAGE, transferred to a PVDF membrane, and detected using anti-HA tag, anti-Flag tag, anti-STAT3, anti-STAT4, anti-p-STAT3, anti-p-STAT4, anti-p-Jak2, anti-Jak2, anti-α-tubulin, and anti-Lamin B1 antibodies. All antibodies were obtained from Cell Signaling Technology.

### Luciferase reporter assay

Cells were cultured in 24-well plates. All plasmids were prepared using a QIAGEN plasmid purification kit. Transient transfection was performed using Lipofectamine 3000 (Invitrogen), and the phRL-SV40 vector (Promega, Madison, WI, USA) was used as a transfection efficiency control. Forty-eight hours after transfection, the cells were lysed, and firefly and Renilla luciferase activities were evaluated using a dual-luciferase reporter assay system (Promega).

### Statistical analysis

Student’s t test was used for two-sample comparisons. One-way ANOVA was used for multiple comparisons. Tukey’s test was used to determine significant differences in the ANOVA data. A value of *p* < 0.05 was defined as significant. All data are presented as the means ± s.d. unless otherwise specified.

## Results

### Enhanced trabecular bone loss and in vivo bone resorption in ALI mice

**Fig. 1:**
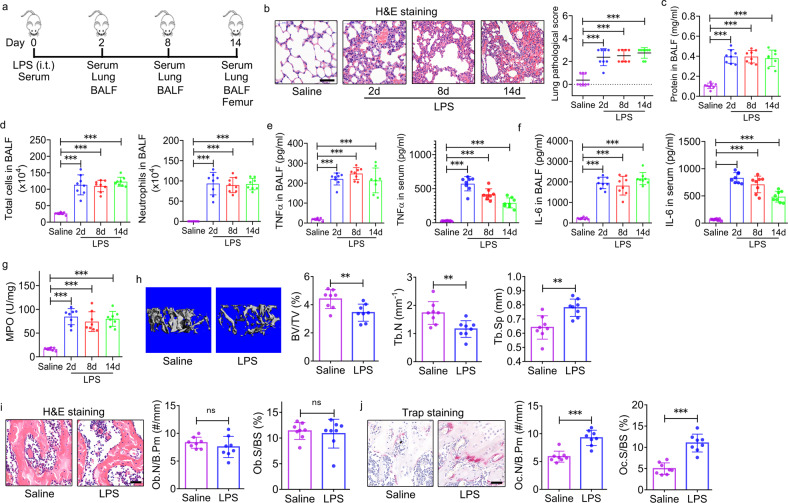
Enhanced trabecular bone loss and in vivo bone resorption in mice with acute lung injury. **a** Schematic diagram showing intratracheal (i.t.) instillation of mice with 5 mg/kg LPS. Mice that were treated with saline were used as negative controls. The day when LPS was administered was defined as “Day 0”. Peripheral blood, lung tissue, and bronchoalveolar lavage fluid (BALF) were harvested on Days 0, 2, 8, and 14. Femurs were collected on Day 14. **b** Lung histology was assessed by H&E staining and quantitative analysis to determine acute lung injury (ALI) (*n* = 8). Scale bar: 50 μm. **c** Total protein concentrations in the BALF of ALI mice determined by the BCA method (*n* = 8). **d** Total cell and neutrophil counts in the BALF of ALI mice (*n* = 8). **e** TNFα levels in the BALF and serum of ALI mice were determined by ELISA (*n* = 8). **f** IL-6 levels in the BALF and serum of ALI mice were determined by ELISA (*n* = 8). **g** Myeloperoxidase (MPO) activity in the lung tissues of ALI mice (*n* = 8). **h** Representative 3D reconstruction images and the trabecular bone volume fraction (BV/TV), trabecular bone number (Tb.N), and trabecular bone separation (Tb.Sp) of the distal femur trabecular bone of ALI mice were determined by micro-CT (*n* = 8). **i** H&E staining and histomorphometric analysis of the osteoblast number (Ob.N/B.Pm) and surface (Ob.S/BS) in the femurs of ALI mice (*n* = 8). Scale bar: 40 μm. **j** TRAP staining and histomorphometric analysis of the osteoclast number (Oc.N/B.Pm) and surface (Oc.S/BS) in the femurs of ALI mice (*n* = 8). Scale bar: 40 μm. The data are representative of three independent experiments. The data are shown as the means ± s.d. **p < 0.01, ***p < 0.001, ns no significance.

To establish the ALI model, we intratracheally instilled *Escherichia coli* LPS in mice. As a control, we similarly instilled mice with saline. Lung tissues and BALF were collected on Days 2, 8, and 14 after LPS administration (Fig. 1a). We observed severe acute alveolar destruction, epithelial cell hyperplasia, and inflammatory infiltration in LPS-treated mice compared to the saline-treated control, which was supported by the semiquantitative analysis of the lung inflammation score (Fig. 1b). Consistent with the histology results, the total protein content (Fig. 1c), total cells, and neutrophil counts (Fig. 1d) were significantly increased compared with those in the saline-treated control group. TNFα (Fig. 1e) and IL-6 (Fig. 1f) levels in the BALF and serum of LPS-treated mice were significantly increased compared with saline-treated mice. Moreover, MPO activity was significantly enhanced in the lung tissues of LPS-treated mice compared with saline-treated mice (Fig. 1g). These data suggested that intratracheal administration of LPS induced ALI in mice.

On Day 14 after LPS administration, femurs were harvested from saline- and LPS-treated mice (Fig. 1a). Micro-CT was performed to assess the bone mass and microstructure of the femur. The 3-D reconstruction images indicated that the trabecular bone of the distal femur was less abundant in LPS-treated mice than in saline-treated mice (Fig. 1h). Consistent with this finding, a significant decrease in the BV/TV and Tb.N, and a significant increase in Tb.Sp in the distal femur were observed in LPS-treated mice compared with saline-treated mice (Fig. 1h). Unlike that in trabecular bone, no differences in the Ct.Ar/Tt.Ar and Ct.Th of the femur mid-shaft were observed between LPS- and saline-treated mice (Supplementary Fig. 1). The micro-CT data revealed significant trabecular bone loss in ALI mice.

In addition to micro-CT scanning, histomorphometric analysis based on H&E and TRAP staining of the femur was performed to evaluate in vivo bone formation and resorption activity in mice. There were no differences in the Ob.N/B.Pm and Ob.S/BS between LPS- and saline-treated mice (Fig. 1i). However, the Oc.N/B.Pm and Oc.S/BS were increased significantly in LPS-treated mice compared with saline-treated mice (Fig. 1j), suggesting enhanced bone resorption in ALI mice.

### CCL12 upregulation is involved in enhanced trabecular bone loss and in vivo bone resorption in ALI mice

**Fig. 2:**
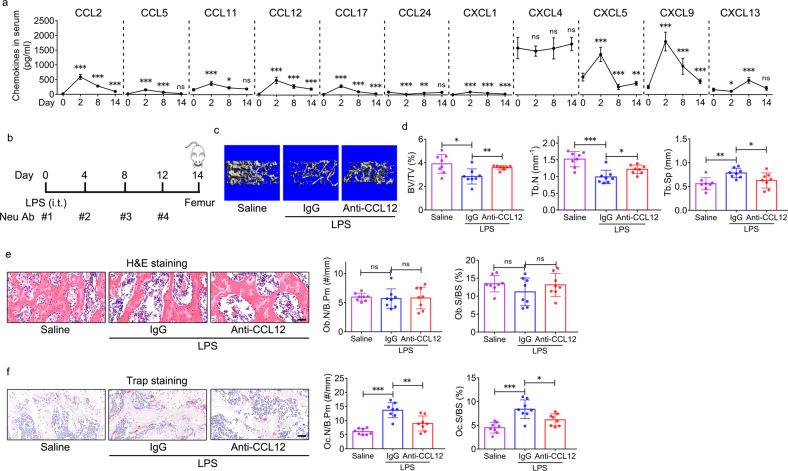
CCL12 upregulation is involved in the enhanced trabecular bone loss and in vivo bone resorption in ALI mice. **a** Chemokine levels in the peripheral blood of ALI mice on Days 0, 2, 8, and 14 after LPS administration were determined by an antibody array (*n* = 6). **b** Schematic diagram of mice treated with 5 mg/kg LPS (i.t.) and 4 mg/kg neutralizing antibodies against CCL12 via the tail vein (*n* = 8). Saline was used as a negative control for LPS. Isotype IgG was used as a negative control for the neutralizing antibody. The day when LPS was administered was defined as “Day 0”. A neutralizing antibody against CCL12 was injected for the first time before LPS administration on Day 0. Then, the antibody was injected every 4 days. Femurs were collected on Day 14. **c** Representative 3D reconstruction images of the distal femur trabecular bone of ALI mice after the administration of the neutralizing antibody against CCL12 were determined by micro-CT. **d** BV/TV, Tb.N, and Tb.Sp of the distal femur in ALI mice after the administration of the neutralizing antibody against CCL12 (*n* = 8). **e** H&E staining and histomorphometric analysis of the Ob.N/B.Pm and Ob.S/BS in the femurs of ALI mice after the administration of the neutralizing antibody against CCL12 (*n* = 8). Scale bar: 40 μm. **f** TRAP staining and histomorphometric analysis of the Oc.N/B.Pm and Oc.S/BS in the femurs of ALI mice after the administration of the neutralizing antibody against CCL12 (*n* = 8). Scale bar: 40 μm. The data are representative of three independent experiments. The data are shown as the means ± s.d. *p < 0.05, **p < 0.01, ***p < 0.001, ns no significance.

Chemokines, which are divided into the major CCL and CXCL subgroups, are essential in regulating cell viability, proliferation, differentiation, and migration^24^. Having observed trabecular bone loss in LPS-treated mice, we investigated the role of chemokines in trabecular bone loss in ALI mice. Peripheral blood was harvested on Days 0, 2, 8, and 14 after LPS administration (Fig. 1a). An antibody array was conducted to investigate the temporal secretion of 11 chemokines, including CCL2, 5, 11, 12, 17, and 24 and CXCL1, 4, 5, 9, and 13, in the serum of LPS- and saline-treated mice. Except for CXCL4, the secretion all the other candidates investigated exhibited significant changes in ALI mice (Fig. 2a). Of these, CCL2, 12, and 17 and CXCL1 and 9 were increased in response to LPS administration compared with baseline (Day 0, without LPS) (Fig. 2a). CCL24 levels declined on Days 2 and 8 and then returned to baseline on Day 14 after LPS administration (Fig. 2a). CCL5 and 11 peaked on Day 2 and then declined and returned to baseline on Day 14 (Fig. 2a). CXCL5 reached its peak on Day 2 and then fell significantly below baseline on Days 8 and 14 (Fig. 2a). CXCL13 levels declined significantly on Day 2 and then increased above baseline on Day 8 and returned to baseline on Day 14 (Fig. 2a).

To study the roles of these chemokines in trabecular bone loss in ALI mice, we used a neutralizing antibody (Neu Ab) to block the in vivo activity of chemokines in ALI mice (Fig. 2b). On Day 14 after LPS administration, femurs were harvested from IgG- and Neu Ab-treated mice (Fig. 2b). Micro-CT showed that Neu Abs against CCL2, CCL5, CCL11, CCL17, and CCL24 did not lead to changes in the BV/TV of the distal femur in ALI mice compared with IgG (Supplementary Fig. 2a, b). A decrease in the BV/TV was observed in ALI mice in response to a Neu Ab against CXCL5 treatment compared with IgG (Supplementary Fig. 2a, b). BV/TV was increased in ALI mice in response to Neu Abs against CCL12 and CXCL1 treatment compared with IgG, and the Neu Ab against CCL12 exerted a more notable effect than CXCL1 (Supplementary Fig. 2a, b) (Fig. 2c, d). Thus, we focused on CCL12 in the subsequent experiments. In addition to the change in BV/TV, a significant decrease in Tb.N, and a significant increase in Tb.Sp in the distal femur were observed in ALI mice in response to anti-Neu Abs against CCL12 compared with IgG (Fig. 2d). No differences in the Ct.Ar/Tt.Ar and Ct.Th of the femur mid-shaft were observed in ALI mice treated with Neu Abs against CCL12 and IgG (Supplementary Fig. 3). Micro-CT data revealed significant inhibition of trabecular bone loss in ALI mice in response to the blockade of CCL12 activity.

Histomorphometric analysis of femurs was performed to evaluate in vivo bone formation and resorption activity in mice. There were no differences in the Ob.N/B.Pm and Ob.S/BS in ALI mice treated with Neu Abs against CCL12 and IgG (Fig. 2e). However, the Oc.N/B.Pm and Oc.S/BS, which were elevated in ALI mice, were decreased significantly in response to Neu Abs against CCL12 compared with IgG (Fig. 2f), suggesting the inhibition of bone resorption in ALI mice in response to the blockade of CCL12 activity.

### Global deletion of CCL12 ameliorates trabecular bone loss in ALI mice

**Fig. 3:**
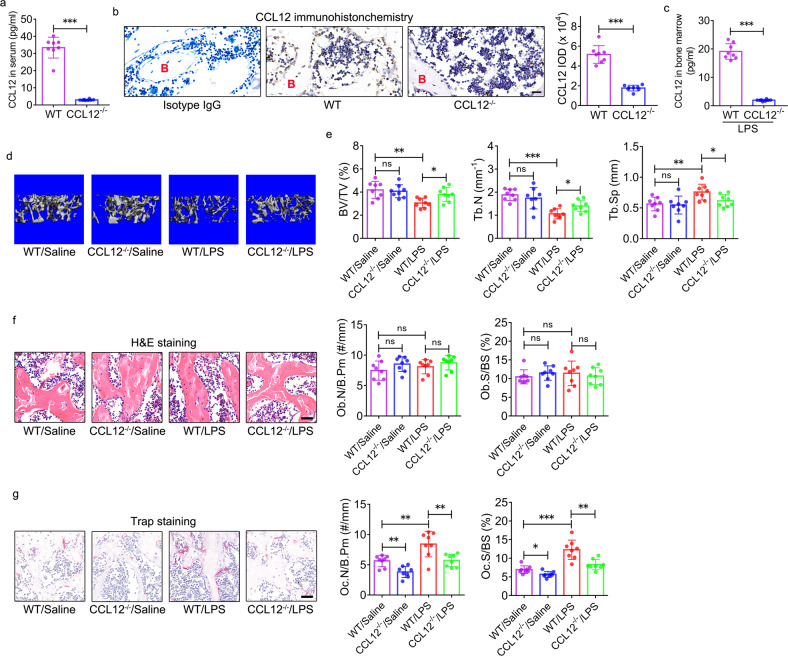
Global deletion of CCL12 ameliorates trabecular bone loss in ALI mice. **a** CCL12 levels in the peripheral blood of wild-type (WT) and CCL12^−/−^ mice were determined by ELISA (*n* = 8). **b**, **c** CCL12 protein expression in the bone marrow of WT and CCL12^−/−^ mice was determined by immunohistochemistry (**b**) and ELISA (**c**) (*n* = 8). B: bone. Scale bar: 20 μm. **d** Representative 3D reconstruction images of the distal femur trabecular bones of WT and CCL12^−/−^ mice were determined by micro-CT. **e** BV/TV, Tb.N, and Tb.Sp of the distal femurs of WT and CCL12^−/−^ mice (*n* = 8). **f** H&E staining and histomorphometric analysis of the Ob.N/B.Pm and Ob.S/BS in the femur (*n* = 8). Scale bar: 40 μm. **g** TRAP staining and histomorphometric analysis of the Oc.N/B.Pm and Oc.S/BS in the femurs of WT and CCL12^−/−^ mice (n = 8). Scale bar: 40 μm. The data are representative of three independent experiments. The data are shown as the means ± s.d. *p < 0.05, **p < 0.01, ***p < 0.001, ns no significance.

To confirm the role of CCL12 in trabecular bone loss in ALI mice, we used global CCL12-knockout mice, in which CCL12 expression was decreased in the serum and bone marrow, as indicated by ELISA (Fig. 3a) and immunohistochemistry (Fig. 3b). Furthermore, when ALI was induced by LPS, there was a very low level of CCL12 in the bone marrow of CCL12^−/−^ mice compared with WT mice (Fig. 3c).

Then, CCL12^−/−^ and WT mice were induced with LPS to develop ALI. Saline was used as a control. Femurs were harvested on Day 14 after LPS or saline administration. Micro-CT was performed to assess the bone mass and microstructure of the femur. The 3-D reconstruction images indicated that there were no changes in the trabecular bone mass of the distal femur between the WT/saline and CCL12^−/−^/saline groups (Fig. 3d). However, trabecular bone mass was greater in CCL12^−/−^/LPS mice than in WT/LPS mice (Fig. 3d). Consistent with this finding, no differences in BV/TV, Tb.N, and Tb.Sp were observed between WT/saline and CCL12^−/−^/saline mice (Fig. 3e). A significant increase in BV/TV and Tb.N, and a significant decrease in Tb.Sp in the distal femur were observed in CCL12^−/−^/LPS mice compared with WT/LPS mice (Fig. 3e). Moreover, global deletion of CCL12 did not change the Ct.Ar/Tt.Ar or Ct.Th of the femur mid-shaft in saline- or LPS-treated mice (Supplementary Fig. 4). Micro-CT analysis revealed significant inhibition of trabecular bone loss in ALI mice in response to global deletion of CCL12.

Histomorphometric analysis of femurs was performed to evaluate in vivo bone formation and resorption activity in mice. Global deletion of CCL12 did not change the Ob.N/B.Pm or Ob.S/BS of the femur in saline- or LPS-treated mice (Fig. 3f). TRAP staining indicated that global deletion of CCL12 led to a significant decline in the Oc.N/B.Pm and Oc.S/BS of the femur in saline- or LPS-treated mice (Fig. 3g), suggesting the inhibition of bone resorption in response to global deletion of CCL12.

In response to global deletion of CCL12, lung inflammation induced by LPS was alleviated on Days 2, 8, and 14 after LPS administration (Supplementary Fig. 5a). Consistent with the histology results, the total protein content (Supplementary Fig. 5b), total cell and neutrophil counts (Supplementary Fig. 5c), and TNFα and IL-6 levels (Supplementary Fig. 5d) in the BALF of CCL12^−/−^/LPS mice were significantly decreased compared with those in the BALF of WT/LPS mice. Moreover, MPO activity was significantly reduced in the lung tissues of CCL12^−/−^/LPS mice compared with WT/LPS mice (Supplementary Fig. 5e). These data suggested that global deletion of CCL12 could relieve lung inflammation in ALI mice.

### CCL12 increases RANKL production in BMSCs via CCR2 in ALI mice

The data thus far suggested the positive role of CCL12 in bone resorption. To elucidate the underlying molecular mechanisms, we isolated bone marrow monocytes (BMMs) and used M-CSF and RANKL to induce BMMs to undergo osteoclastic differentiation in vitro in with the presence of recombinant CCL12 (rCCL12). TRAP staining and quantification revealed no difference in osteoclast formation with or without rCCL12 treatment (Supplementary Fig. 6a). qRT‒PCR analysis of key transcription factors and marker genes associated with osteoclast differentiation, including c-fos, NFATc1, cathepsin K, β3-integrin, DC-STAMP, and ATP6v0d2, revealed the same pattern as TRAP staining (Supplementary Fig. 6b, c). These data suggested that CCL12 alone could not directly activate osteoclast formation.

**Fig. 4:**
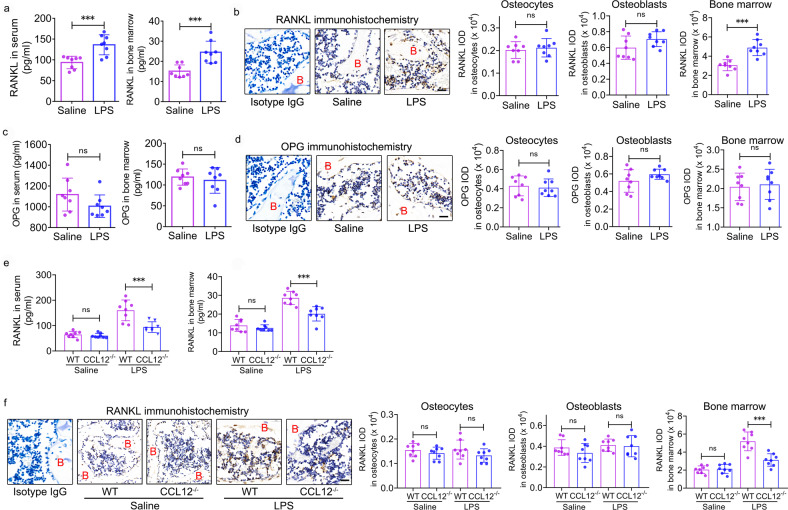
CCL12 enhances RANKL levels in the bone marrow of ALI mice. **a** RANKL levels in peripheral blood and bone marrow of ALI mice were determined by ELISA (*n* = 8). **b** RANKL protein expression in the femur osteocytes, osteoblasts, and bone marrow of ALI mice was determined by immunohistochemistry (*n* = 8). B: bone. Scale bar: 20 μm. **c** OPG levels in the peripheral blood and bone marrow of ALI mice were determined by ELISA (*n* = 8). **d** OPG protein expression in the femur osteocytes, osteoblasts, and bone marrow of ALI mice was determined by immunohistochemistry (*n* = 8). B: bone. Scale bar: 20 μm. **e** RANKL levels in the peripheral blood and bone marrow of ALI mice with global deletion of CCL12 were determined by ELISA (*n* = 8). **f** RANKL protein expression in the femur osteocytes, osteoblasts, and bone marrow of ALI mice with global deletion of CCL12 was determined by immunohistochemistry (*n* = 8). B: bone. Scale bar: 20 μm. The data are representative of three independent experiments. The data are shown as the means ± s.d. **p < 0.01, ***p < 0.001, ns no significance.

Therefore, we hypothesized that CCL12 could activate osteoclast formation and bone resorption through indirect means. RANKL/OPG is one of the most important signaling pathways that modulates osteoclast differentiation, formation, and bone resorption in the skeletal system^25^. RANKL levels were significantly elevated in the serum and bone marrow of LPS-treated mice compared with saline-treated mice (Fig. 4a). Immunohistochemical analysis of femur bone marrow showed that RANKL protein expression in osteocytes and osteoblasts remained unchanged between saline- and LPS-treated mice (Fig. 4b). In contrast, RANKL protein expression in bone marrow was significantly upregulated in LPS-treated mice compared with saline-treated mice (Fig. 4b), which supported the ELISA data. The ELISA (Fig. 4c) and immunohistochemistry (Fig. 4d) data revealed no differences in OPG levels in the serum, osteocytes, osteoblasts, and bone marrow between LPS- and saline-treated mice. Furthermore, global deletion of CCL12 reduced RANKL levels in the serum and bone marrow of LPS-treated mice (Fig. 4e, f). These data suggested that CCL12 exerted positive effects on RANKL production in ALI mice.

RANKL is mainly produced by BMSCs, osteoblasts, and osteocytes in the skeletal system^26^. To clarify the source of RANKL in ALI mice, we isolated BMSCs, osteoblasts, and osteocytes from LPS- and saline-treated mice. Flow cytometry was used to investigate RANKL expression in these cells. No differences were observed in osteoblasts (Supplementary Fig. 7a) and osteocytes (Supplementary Fig. 7b) from LPS- and saline-treated mice. Moreover, rCCL12 failed to change RANKL mRNA and protein expression levels in primary osteoblasts, the 3T3-E1 cell line, primary osteocytes, and the MLO-Y4 cell line (Supplementary Fig. 7c–f).

**Fig. 5:**
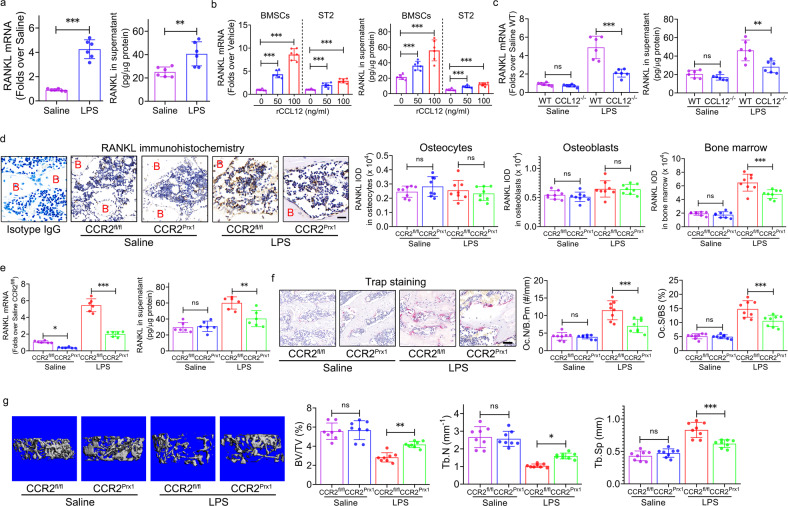
CCL12 increases RANKL production in BMSCs via CCR2 in ALI mice. **a** RANKL mRNA and protein expression in RANKL^+^CCR2^+^ BMSCs from ALI mice (*n* = 8). **b** RANKL mRNA and protein expression in primary BMSCs and ST2 cells cultured with 0, 50, and 100 ng/ml recombinant CCL12 for 48 h in vitro (*n* = 8). **c** RANKL mRNA and protein expression in RANKL^+^CCR2^+^ BMSCs from ALI mice with global deletion of CCL12 (*n* = 8). **d** RANKL protein expression in the femur osteocytes, osteoblasts, and bone marrow of ALI mice with conditional deletion of CCR2 in BMSCs was determined by immunohistochemistry (*n* = 8). B: bone. Scale bar: 20 μm. **e** RANKL mRNA and protein expression in BMSCs with CCR2 deletion from ALI mice was determined by flow cytometry (*n* = 8). **f** TRAP staining and histomorphometric analysis of the Oc.N/B.Pm and Oc.S/BS in the femurs of ALI mice with conditional deletion of CCR2 in BMSCs (*n* = 8). Scale bar: 40 μm. **g** Representative 3D reconstruction images and the BV/TV, Tb.N, and Tb.Sp of the distal femur trabecular bones of ALI mice with conditional deletion of CCR2 in BMSCs were determined by micro-CT (*n* = 8). α-Tubulin was used as an internal control for qPCR. The data are representative of three independent experiments. The data are shown as the means ± s.d. *p < 0.05, **p < 0.01, ***p < 0.001, ns no significance.

Flow cytometric analysis of the general mesenchymal surface markers CD44, 73, 90, and 105 was used to obtain BMSCs from bone marrow (Supplementary Fig. 8a). The results revealed that RANKL expression was significantly elevated in BMSCs from LPS-treated mice compared with those from saline-treated mice (Supplementary Fig. 8b), and CCR2 expression remained unchanged in BMSCs from saline- and LPS-treated mice (Supplementary Fig. 8c). RANKL^+^ CCR2^+^ BMSCs were further sorted by flow cytometry (Supplementary Fig. 8d). RANKL mRNA expression and secretion levels were significantly elevated in the BMSC population from LPS-treated mice compared with the population from saline-treated mice (Fig. 5a). Moreover, RANKL mRNA and protein expression were upregulated by rCCL12 treatment in primary RANKL^+^ CCR2^+^ BMSCs and the ST2 cell line (Fig. 5b). Global deletion of CCL12 inhibited RANKL mRNA and protein expression in total BMSCs (Supplementary Fig. 8e) and RANKL^+^ CCR2^+^ BMSCs from LPS-treated mice (Fig. 5c).

CCL chemokines bind to CCR receptors^27^. The receptor for CCL12 is CCR2^27^. CCR2^Prx1^ conditional knockout mice were generated by breeding CCR2^fl/fl^ mice with Prx1-Cre transgenic mice, in which CCR2 was deleted from BMSCs. A significant decrease in RANKL expression was observed in the bone marrow of LPS-treated CCR2^Prx1^ mice compared with CCR2^fl/fl^ mice (Fig. 5d), and RANKL expression remained unchanged in osteocytes and osteoblasts from CCR2^Prx1^ and CCR2^fl/fl^ mice (Fig. 5d). Consistent with the immunohistochemistry results, RANKL expression was significantly reduced in BMSCs from LPS-treated CCR2^Prx1^ mice compared with CCR2^fl/fl^ mice (Supplementary Fig. 8f). Moreover, RANKL^+^CCR2^−/−^ BMSCs that were cultured in vitro showed decreased levels of RANKL mRNA and protein expression (Fig. 5e).

Histomorphometric analysis of femurs was performed to evaluate the in vivo bone resorption activity of LPS-treated CCR2^Prx1^ mice. Conditional deletion of CCL12 from BMSCs led to a significant decline in the Oc.N/B.Pm and Oc.S/BS of the femur in LPS-treated mice (Fig. 5f). Micro-CT 3-D reconstruction images indicated that the trabecular bone mass of the distal femur was greater in LPS-treated CCR2^Prx1^ mice than in LPS-treated CCR2^fl/fl^ mice (Fig. 5g). Consistent with this finding, a significant increase in the BV/TV and Tb.N, and a significant decrease in Tb.Sp in the distal femur were observed in CCR2^Prx1^/LPS mice compared with CCR2^fl/fl^/LPS mice (Fig. 5g). Conditional deletion of CCR2 from BMSCs did not change the Ct.Ar/Tt.Ar or Ct.Th of the femur mid-shaft in saline- or LPS-treated mice (Supplementary Fig. 9). Micro-CT analysis revealed significant inhibition of trabecular bone loss in ALI mice in response to conditional deletion of CCR2 from BMSCs.

### The Jak2/STAT4 axis in CCL12-mediated activation of RANKL expression in BMSCs

**Fig. 6:**
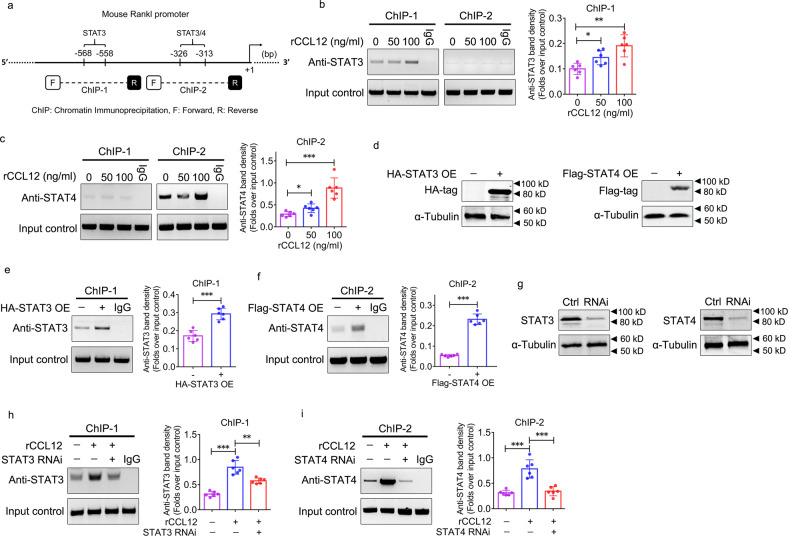
STAT3 and STAT4 binding to the RANKL promoter during CCL12-mediated activation of RANKL expression in BMSCs. **a** Diagram of the mouse RANKL promoter. Analysis of transcription factor binding sites in the RANKL promoter revealed putative STAT3 and STAT4 binding sites around the transcription start site. **b** STAT3 binding to the ChIP-1 and ChIP-2 regions within the RANKL promoter in BMSCs in response to 0, 50, and 100 ng/ml recombinant CCL12 treatment for 24 h, as determined by ChIP. **c** STAT4 binding to the ChIP-1 and ChIP-2 regions within the RANKL promoter in BMSCs in response to 0, 50, and 100 ng/ml recombinant CCL12 treatment for 24 h, as determined by ChIP. **d** Lentivirus-mediated stable overexpression (OE) of STAT3 with an HA tag and STAT4 with a Flag tag in BMSC cultures was determined by western blotting. **e** STAT3 binding to the ChIP-1 region within the RANKL promoter in BMSCs with STAT3 overexpression was determined by ChIP. **f** STAT4 binding to the ChIP-2 region within the RANKL promoter in BMSCs with STAT4 overexpression was determined by ChIP. **g** Lentivirus-mediated stable knockdown of STAT3 and STAT4 in BMSC cultures was determined by western blotting. **h** STAT3 binding to the ChIP-1 region within the RANKL promoter in BMSCs treated with recombinant CCL12 for 24 h and/or STAT3 knockdown was determined by ChIP. **i** STAT4 binding to the ChIP-2 region within the RANKL promoter in BMSCs treated with recombinant CCL12 for 24 h and/or STAT4 knockdown was determined by ChIP. Isotype IgG was used as a negative control in the ChIP assay. α-Tubulin was used as a loading control for western blotting. The data are representative of three independent experiments. The data are shown as the means ± s.d. *p < 0.05, **p < 0.01, ***p < 0.001.

To elucidate the molecular mechanisms underlying CCL12-mediated activation of RANKL expression in BMSCs, we screened the mouse RANKL proximal promoter (−1005 bp to +156 bp) using JASPAR (http://jaspar.genereg.net/) for putative transcription factor binding sites, and the results revealed a putative STAT3 binding site (−568 bp to −558 bp) and a putative STAT4 binding site (−326 bp to −313 bp) (Supplementary Fig. 10) (Fig. 6a). ChIP was performed using primers for to amplify the RANKL promoter surrounding STAT3 (defined as “ChIP-1”) and STAT4 (defined as “ChIP-2”) to investigate the binding of STAT3 and STAT4 to the RANKL promoter in BMSCs treated with rCCL12. STAT3 did not bind to the ChIP-2 region but did bind to the ChIP-1 region in the RANKL promoter, and its binding ability was increased in response to rCCL12 treatment (Fig. 6b). Accordingly, STAT4 did not bind to the ChIP-1 region but did bind to the ChIP-2 region in the RANKL promoter, and its binding ability was increased in response to rCCL12 treatment (Fig. 6c). STAT3 and STAT4 were overexpressed in BMSCs by lentiviral vectors (Fig. 6d). The ChIP data revealed an increase in STAT3 binding to the ChIP-1 region in response to STAT3 overexpression (Fig. 6e) and STAT4 binding to the ChIP-2 region in response to STAT4 overexpression (Fig. 6f). Furthermore, STAT3 and STAT4 expression was silenced in BMSCs by lentivirus-mediated RNAi (Fig. 6g). In response to STAT3 and STAT4 knockdown, significant inhibition of STAT3 binding to the ChIP-1 region (Fig. 6h) and STAT4 binding to the ChIP-2 region (Fig. 6i) in the RANKL promoter was observed, and this effect was enhanced by rCCL12.

**Fig. 7:**
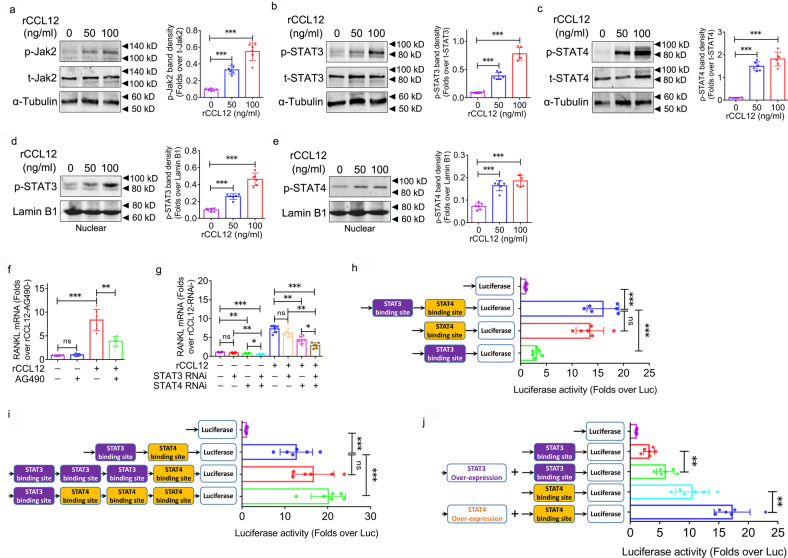
Jak2/STAT4 axis in CCL12-mediated activation of RANKL expression in BMSCs. **a** Phosphorylated Jak2 protein levels in BMSCs in response to 0, 50, and 100 ng/ml recombinant CCL12 treatment for 6 h were determined by western blotting. **b** Phosphorylated STAT3 protein levels in BMSCs in response to 0, 50, and 100 ng/ml recombinant CCL12 treatment for 6 h were determined by western blotting. **c** Phosphorylated STAT4 protein levels in BMSCs in response to 0, 50, and 100 ng/ml recombinant CCL12 treatment for 6 h were determined by western blotting. **d** Phosphorylated STAT3 protein levels in the nuclear fraction of BMSCs in response to 0, 50, and 100 ng/ml recombinant CCL12 treatment for 6 h were determined by western blotting. **e** Phosphorylated STAT4 protein levels in the nuclear fraction of BMSCs in response to 0, 50, and 100 ng/ml recombinant CCL12 treatment for 6 h were determined by western blotting. **f** RANKL mRNA expression in BMSCs in response to recombinant CCL12 (100 ng/ml) and/or AG490 (50 μM) treatment for 48 h (*n* = 8). **g** RANKL mRNA expression in BMSCs in response to recombinant CCL12 (100 ng/ml) treatment for 48 h and/or STAT3/4 knockdown (*n* = 8). **h** Luciferase activity of RANKL promoter deletion mutant-driven luciferase reporter gene vectors, including Luc (empty vector as negative control), −1005 bp/+156 bp (containing putative STAT3 and STAT4 binding sites), −454 bp/+156 bp (containing putative STAT4 binding site), and −1005 bp/−429 bp (containing putative STAT3 binding site), in BMSCs treated with recombinant CCL12 for 48 h (*n* = 8). **i** Luciferase activity of RANKL promoter deletion mutant-driven luciferase reporter gene vectors, including Luc, STAT3 + STAT4 binding site, 3×STAT3 + STAT4 binding site, and STAT3 + 3×STAT4 binding site, in BMSCs treated with recombinant CCL12 for 48 h (*n* = 8). **j** Luciferase activity of RANKL promoter deletion mutant-driven luciferase reporter gene vectors with and without pcDNA3.1-STAT3/4 vector cotransfection in BMSCs treated with recombinant CCL12 for 48 h (*n* = 8). α-Tubulin was used as an internal control for qPCR and western blot analysis of total proteins. Lamin B1 was used as a loading control for western blot analysis of nuclear proteins. The Renilla luciferase vector was used as a control for transfection efficiency. The data are representative of three independent experiments. The data are shown as the means ± s.d. *p < 0.05, **p < 0.01, ***p < 0.001, ns no significance.

Having observed the binding of STAT3 and STAT4 to the RANKL promoter in BMSCs, we then evaluated their contributions to RANKL expression induced by CCL12 in BMSCs. CCL12 increased the phosphorylation of Jak2, a kinase that is upstream of STAT3 and STAT4, in a dose-dependent manner in BMSCs (Fig. 7a). Accordingly, the phosphorylation of STAT3 (Fig. 7b) and STAT4 (Fig. 7c) was also increased in response to rCCL12 treatment. Moreover, CCL12 enhanced the levels of phosphorylated STAT3 (Fig. 7d) and STAT4 (Fig. 7e) in the nucleus of BMSCs. When AG490 was used to inhibit Jak2, rCCL12-induced RANKL mRNA expression was significantly inhibited (Fig. 7f). Likewise, CCL12-mediated activation of RANKL mRNA expression was significantly inhibited by siRNA-mediated STAT3 and STAT4 knockdown (Fig. 7g). It should be noted that STAT3 knockdown alone failed to inhibit RANKL mRNA expression, but STAT4 knockdown alone did (Fig. 7g), regardless of rCCL12 treatment, suggesting a much larger role of STAT4 than STAT3 in CCL12-mediated activation of RANKL expression.

We amplified the mouse RANKL proximal promoter (−1005 bp to +156 bp) and generated two mutants with deletion of the STAT3 or STAT4 binding site. The various RANKL promoter fragments were cloned into luciferase reporter gene vectors, and a transient reporter assay was performed on BMSCs after 48 h of rCCL12 treatment. The luciferase activity of the complete RANKL promoter-driven construct exhibited significant increases compared with the empty luciferase vector (Fig. 7h). When the STAT3 binding site was deleted, no change was observed in luciferase activity (Fig. 7h), suggesting the dispensable role of the STAT3 binding site in CCL12-mediated activation of RANKL expression. However, there was a marked decrease in the activity of the STAT4 binding site deletion mutant-driven construct compared with the complete promoter-driven construct (Fig. 7h), highlighting the essential contribution of the STAT4 binding site to CCL12-mediated activation of RANKL expression. Consistent with these data, when three STAT3 binding sites were cloned upstream of the STAT4 binding site, this mutant-driven construct failed to induce any change in luciferase activity compared with the complete promoter-driven construct (Fig. 7i). In contrast, three STAT4 binding sites cloned downstream of the STAT3 binding site enhanced luciferase activity compared with the complete promoter-driven construct (Fig. 7i). Furthermore, we constructed plasmid vectors to overexpress STAT3 and STAT4 and cotransfected these vectors with STAT3- or STAT4-driven constructs into BMSCs. The activity of the STAT3- or STAT4-driven construct was enhanced in response to STAT3 or STAT4 overexpression, respectively (Fig. 7j). However, the effect of STAT4 overexpression was more significant than that of STAT3 overexpression (Fig. 7j), confirming the more important contribution of the STAT4 binding site than the STAT3 binding site to CCL12-mediated activation of RANKL expression.

## Discussion

The current study sheds light on the bone phenotypes of ALI mice. Trabecular bone loss was observed in the distal femurs of LPS-treated mice. During ALI, CCL12 accumulates in the peripheral blood and bone marrow. In vivo blockade of CCL12 via a neutralizing antibody and global knockout in mice ameliorated trabecular bone loss, suggesting the negative role of CCL12 in bone mass of ALI mice. Furthermore, we found that CCL12 enhanced bone resorption by stimulating RANKL production in BMSCs, and the Jak2/STAT4 axis played an important role in this process.

In this study, inhaled LPS challenge resulted in airway neutrophil influx, inflammatory mediator release, and lung inflammatory infiltration. These data are supported by several previous studies^17,28,29^. Trabecular bone loss was observed on Day 14 after LPS administration. In the first animal model examining a lung inflammatory-bone loss relationship, the authors demonstrated that 3 weeks of exposure to inhaled environmental inflammatory agents, including LPS, gram-positive peptidoglycan, and complex organic dust extract, induced neutrophilic airway disease with resultant loss of bone quantity and quality^30^. Other studies demonstrated that low-dose and repetitive LPS administration for 3 weeks could lead to bone loss^31,32^. To the best of our knowledge, the current study is the first in vivo animal study to demonstrate that a single administration of LPS was sufficient to induce trabecular bone loss on Day 14 after LPS administration. These data provide important insights into the animal model of lung inflammation-induced bone loss to help guide future preclinical studies examining the airway inflammatory-bone deterioration disease axis.

In the current study, bone histomorphometry analysis suggested that LPS-induced trabecular bone loss did not result from the attenuation of in vivo bone formation but from enhanced bone resorption. Bone tissue maintains homeostasis through the synergy between osteoblasts (bone-forming cells) and osteoclasts (bone-resorbing cells). Osteoclasts are differentiated from BMMs^25^. In rheumatoid arthritis, localized and systemic inflammation leads to an imbalance in this equilibrium, which is known to be secondary to an increase in osteoclast activity^33^. In addition, LPS has been shown to directly mediate inflammatory skeletal diseases, including osteomyelitis, arthritis, and periodontitis^34,35^. Although M-CSF and RANKL are necessary for osteoclastogenesis, studies have shown that LPS influences osteoclastogenesis in a dose-dependent manner^35^. Moreover, LPS, which is recognized by TLR4, triggers an inflammatory cascade via the activation of NF-кB and MAPK, resulting in bone resorption^36^. Our findings of inhalation-induced lung inflammation and subsequent trabecular bone loss suggest exists that LPS escaped from lung vessels into systemic circulation to directly affect skeletal health. However, LPS could be transiently detected in the peripheral blood only within the first 6 h after administration and was not be detected until Day 14 after administration (data not shown), suggesting that LPS is unlikely to activate bone resorption directly. We hypothesized that lung injury-induced inflammatory mediators may indirectly activate osteoclasts or disrupt the osteoclast-osteoblast equilibrium.

An antibody array was used to screen the levels of chemokines in the peripheral blood during ALI. We found that the levels of most of the candidates were significantly elevated in response to LPS administration. A neutralizing antibody was used and inhibited trabecular bone loss in response to LPS administration. Of these candidates, CCL12 exhibited the most potent effects, suggesting its role in the trabecular bone loss induced by lung inflammation. When CCL12 was systemically deleted in vivo, bone resorption and trabecular bone loss were significantly inhibited in mice, which confirmed the involvement of CCL12 in lung inflammation-induced bone disease. Few studies have reported the role of CCL12 in the skeleton. To the best of our knowledge, this is the first report to demonstrate that CCL12 plays a positive role in bone resorption and trabecular bone loss during ALI. Moreover, lung histological evaluation, protein concentration analysis, inflammatory cell counts, and TNFα and IL-6 levels in BALF suggested that LPS-induced lung inflammation was alleviated when CCL12 was systemically deleted in vivo, which suggested the potential regulatory role of CCL12 in ALI induced by LPS. The data from the antibody array revealed a significant increase in serum CCL12 levels, and we hypothesize that CCL12, which is induced by LPS, can attract inflammatory cells into the lung, mediate inflammation and ultimately lead to ALI. Therefore, when CCL12 was globally deleted in vivo, lung inflammation was alleviated, including a reduction in protein concentrations, a decrease in inflammatory cell numbers, and the inhibition of TNFα and IL-6 secretion in BALF. Further research is required to validate this hypothesis in the future.

Due to the CCL12-induced increase bone resorption, we hypothesized that there were two potential mechanisms: CCL12 directly stimulated osteoclast differentiation in BMMs or CCL12 facilitated bone resorption through indirect means, such as activating another factor or signaling pathway. In the current study, the possibility that CCL12 directly stimulated osteoclast differentiation in BMMs was ruled out because recombinant CCL12 exerted no effects on osteoclast differentiation in BMMs in vitro, as shown by TRAP staining and the mRNA expression of key transcription factors and marker genes associated with osteoclast differentiation. Therefore, we hypothesized that CCL12 stimulated bone resorption mainly through indirect means in the current study.

Osteoclasts are derived from mononuclear precursors in the myeloid lineage of hematopoietic cells. M-CSF expression by osteoblastic stromal cells is needed for progenitor cells to differentiate into osteoclasts, but M-CSF alone is unable to complete this process. RANKL, RANK, and OPG are essential molecules involved in osteoclast development and bone remodeling^37^. These factors are highly expressed in osteoblasts, osteocytes, and primitive mesenchymal cells, and RANKL is essential for the differentiation of osteoclast precursors and activation of mature osteoclasts. OPG functions as a soluble decoy receptor of RANKL and competes with RANK for RANKL binding. In the current study, we found that RANKL levels were significantly elevated in the serum and bone marrow of LPS-treated mice compared with saline-treated mice. However, no difference in OPG levels was observed, suggesting the involvement of RANKL in the increase in bone resorption in ALI mice. Furthermore, global deletion of CCL12 reduced RANKL levels in the serum and bone marrow of LPS-treated mice, which suggested that CCL12 exerted positive effects on RANKL production in ALI mice.

Moreover, the source of CCL12-induced RANKL production was investigated in the current study. RANKL is highly expressed in lymph nodes, the thymus, mammary glands, and the lung and is expressed at low levels in a variety of other tissues, including the spleen, synovial cells, and bone marrow^38^. In the skeletal system, RANKL is mainly produced by BMSCs, osteoblasts, and osteocytes^26^. We isolated BMSCs, osteoblasts, and osteocytes from LPS- and saline-treated mice. No differences in RANKL expression were observed in osteoblasts and osteocytes from LPS- and saline-treated mice. Moreover, rCCL12 failed to change RANKL mRNA and protein expression in primary osteoblasts, the 3T3-E1 cell line, primary osteocytes, and the MLO-Y4 cell line. RANKL expression was significantly elevated in BMSCs from LPS-treated mice compared with saline-treated mice. Moreover, rCCL12 upregulated RANKL mRNA and protein expression in primary BMSCs and the ST2 cell line. Global deletion of CCL12 inhibited RANKL production in BMSCs from LPS-treated mice. These data suggested that CCL12 promoted bone resorption mainly by stimulating RANKL production in BMSCs. Interestingly, a previous study showed that RANKL induced the expression of CCL12 in RAW264.7 cells^39^. Together with our data from the current study, it is possible that there is a positive feedback loop between CCL12 and RANKL in which these factors can activate each other and promote osteoclast differentiation in combination. Future research may be needed to confirm the presence of this positive feedback loop between CCL12 and RANKL and elucidate the underlying molecular mechanisms.

To elucidate the mechanisms underlying CCL12-mediated activation of RANKL expression in BMSCs, we analyzed the RANKL proximal promoter, in which one STAT3 and one STAT4 binding site were observed. Although ChIP assays confirmed the binding of both STAT3 and STAT4 to the RANKL promoter, further functional experiments revealed that the STAT3 binding site and STAT4 binding site did not contribute equally to CCL12-mediated activation of RANKL expression. The STAT4 binding site was indispensable, and the STAT3 binding site played a minor role in CCL12-mediated activation of RANKL expression. RANKL is one of the major outputs of the JAK/STAT signaling pathway^40^. Sundaram et al. showed that the STAT binding motif was −8 kb upstream of the start codon of the RANKL gene and that STAT-1 binds to this region in response to FGF-2 stimulation^41^. The binding of STAT3, which is one of the major transcription factor targets of gp130-mediated activation of cytokines, to the mouse RANKL locus has been observed in osteoblasts after OSM treatment^42,43^. Srivastava et al. showed that STAT5 regulates the proximal mouse RANKL gene promoter^44^. Sundaram et al. have shown that TRAIL induces p-STAT-6 expression, nuclear translocation, and binding to the hRANKL gene distal promoter region to activate RANKL expression^45^. Collectively, STAT family members show differential cytokine responses and functional significance with respect to RANKL expression. However, there has been no report concerning the role of STAT4 in RANKL expression. To the best of our knowledge, the current study is the first to report that the Jak2/STAT4 axis plays an essential role in CCL12-induced RANKL expression.

There are potential limitations to our study. First, age has already been shown to be an important factor that modulates the lung-bone inflammatory axis in mice. Aged mice (age 12–14 months) have dramatically reduced BMD, bone volume, and trabecular numbers compared to their younger counterparts (age 7–9 weeks) in the absence of airway inflammatory challenges^18^. The current study used young mice (8–10 weeks old) rather than aged mice. We believe that research on the role of CCL12 in lung inflammation-induced trabecular bone loss in aged mice will be informative. Second, the source of the high levels of CCL12 in the bone marrow of mice is an important but difficult question to answer, as the bone marrow is a very complex microenvironment containing numerous cell types. It would be extremely difficult to isolate all cell types in the bone marrow and compare CCL12 expression between LPS- and saline-treated mice. To uncover which cell type is the most important in regulating the expression of CCL12 in the bone marrow, further research with conditional knockout mice using promoters expressed by specific cell types (e.g., HSCs, BMSCs) and differentiated cells (e.g., adipocytes) should be conducted.

**Fig. 8:**
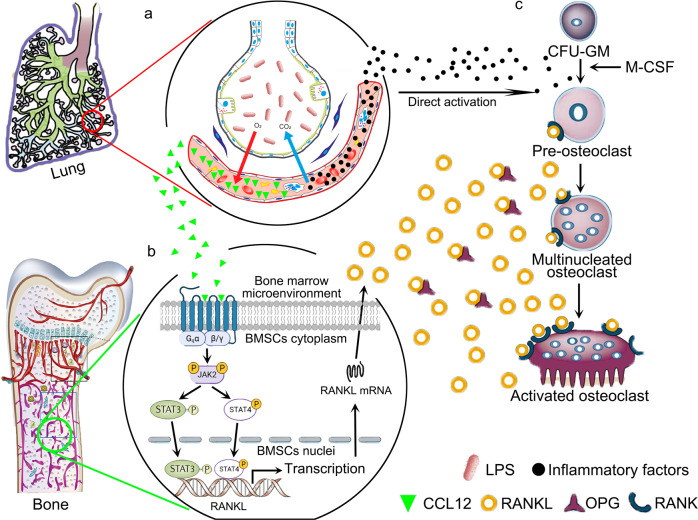
Diagram of the role of CCL12 in trabecular bone loss in ALI mice. LPS induces acute lung injury, and CCL12 levels are significantly elevated in the peripheral blood (**a**) and bone marrow (**b**) in response to inflammation. In bone marrow, CCL12 binds to CCR2 on the cell membrane of BMSCs to activate RANKL transcription through the Jak2/STAT4 axis (**b**). RANKL promotes osteoclastic differentiation in bone marrow monocytes (**c**), which results in enhanced bone resorption activity and trabecular bone loss in acute lung injury.

In summary, this study demonstrated that in addition to direct osteoclastogenesis activation by several inflammatory factors, such as TNFα and IL-6, there was a pathway through which osteoclastogenesis was indirectly activated, and CCL12 accumulated in the bone marrow of LPS-treated mice (Fig. 8a) and stimulated RANKL production via the CCR2/Jak2/STAT4 axis in BMSCs (Fig. 8b). As a result, bone resorption was enhanced, and trabecular bone loss was induced (Fig. 8c).

## Supplementary information

Supplementary data

## Data Availability

The data that support the findings of this study are openly available in Mendeley at 10.17632/h5bw57k744.1.
