# Supplementary material for: CCL12 induces trabecular bone loss by stimulating RANKL production in BMSCs during acute lung injury
Source: Exp Mol Med. 2023 Apr 3;55(4):818–30. doi: 10.1038/s12276-023-00970-w (PMC10167364; doi:10.1038/s12276-023-00970-w)
Supplement: Supplementary file 1 — Supplementary data [file 12276_2023_970_MOESM1_ESM.pdf]

Supplementary data

Materials and methods

Myeloperoxidase (MPO) activity

On day 2 after LPS administration, mice were sacrificed and lungs were removed. Lung samples were weighed and stored at -80 °C. MPO activity was determined with a MPO colorimetric activity assay kit (MAK069; Sigma-Aldrich), according to manufacturer's instructions.

Administration of *in vivo* neutralization antibody against chemokines family members

Mice were treated with 5 mg/kg LPS (i.t.) and 4 mg/kg neutralization antibody against CCL12 via tail vein. Saline was used as negative control for LPS. And isotype IgG was used as negative control for neutralization antibody. The day when LPS was administrated was defined as "Day 0". Neutralization antibody against CCL2, 5, 11, 12, 17, and 24, CXCL1, 5, 9, and 13 was injected for the first time before LPS administration on Day 0. Then antibody was injected every 4 days. All the antibodies were from Thermo Fisher Scientific except for CCL17 (R&D systems).

Flow cytometry

About 1×10^6 cells were incubated on ice for 30 min with goat serum. Subsequently, the cells were stained for 30 min at 4 °C with anti-RANKL antibody (Thermo Fisher Scientific). Isotype-matched antibodies (Thermo Fisher Scientific) were used to rule out non-specific staining of the cells. The labeled cells were thoroughly washed with PBS and analyzed on a BD Calibur machine (BD Biosciences, USA) using the Cell quest as data acquisition software.

Lentivirus vectors

Plasmid vectors loading coding sequences (CDS) and siRNA sequences of mouse STAT3 and STAT4 for lentivirus packaging were from Genecopoeia (Rockville, MD, USA). 293T cells were plated and the transfection complex was added to the culture medium at 70-80% confluence of 293T cells. Then the cells

were incubated in a CO_2 incubator at 37 °C for 48 h and the medium was then collected. Lentivirus titer was evaluated by Lenti-X p24 Rapid titer kit (Clontech, Mountain View, CA, USA).

Osteoclast differentiation assay

Animals were euthanized using isoflurane inhalation anesthesia followed by cervical dislocation. The femur and tibiae of mice were washed with serum-free α-MEM. Bone marrow cells were incubated with α-MEM supplemented with 10% FBS, penicillin (100 U/ml) and streptomycin (100 mg/ml) for 24 h. Non-adherent cells were harvested, and induced to bone marrow monocytes (BMMs) using M-CSF (20 ng/ml). Cells were cultured in the humidified atmosphere at 37 °C and 5% CO_2.

BMMs were incubated in 48-well plate in α-MEM containing M-CSF (20 ng/ml) plus RANKL (100 ng/ml) to form mature osteoclasts. Osteoclasts differentiation was observed by Tartrate resistant acid phosphatase (TRAP) staining. TRAP+ cells with more than three nuclei were considered as mature osteoclasts. TRAP+ cells with more than five nuclei were considered as large osteoclasts.

Osteoblasts isolation and *in vitro* culture

Osteoblasts from mice was isolated and cultured *in vitro* according to the previous protocol (Chevalier C, et al. STAR Protoc. 2021.).

Osteocytes isolation and *in vitro* culture

Osteocytes from mice was isolated and cultured *in vitro* according to the previous protocol (Shah KM, et al. Bonekey Rep. 2016.).

Supplementary Table1: Primer sequences used in qRT-PCR

Gene	Primer sequences (5’-3’)	Product length (bp)
RANKL	TCGGGAAGCGTACCTACAGA (F)	247
	CCCCAAAGTACGTCGCATCT (R)	
c-fos	TGTTCCTGGCAATAGCGTGT (F)	170
	TCAGACCACCTCGACAATGC (R)	
NFATc1	GAGACCGAGAGGCTCCGAAC (F)	209
	CCTCTCCTTTGCCGACACG (R)	
Cathepsin K	CTCCAGTCAAGAACCAGGGC (F)	196
	CCGTTCTGCTGCACGTATTG (R)	
β3-integrin	GGACAACTCTGGGCCGCTC (F)	181
	CCTTCAGGTTACATCGGGGTG (R)	
DC-STAMP	TTTCCACGAAGCCCTAGCTG (F)	233
	GCGTTCCTACCTTCACGGAG (R)	
ATP6v0d2	TGGTTCGAGGATGCAAAGCC (F)	112
	TTGCCATAGTCCGTGGTCTG (R)	
α-Tubulin	TGTGGATTCTGTGGAAGGCG (F)	149
	AGCACACATTGCCACATACAAA (R)	

F: forward, R: reverse

Supplementary Table2: Primer sequences used in ChIP-qPCR

Gene	Region	Primer sequences (5’-3’)	Product length (bp)
RANKL	ChIP-1	CTCAGCATAAGGCTTTCTTA (F)	229
		TCAAATGGGGCAGATGCTCC (R)	
	ChIP-2	GAGGCTATATTTGGAGGGAT (F)	271
		TCTTCGCTCTCAGGGACCCG (R)	

F: forward, R: reverse

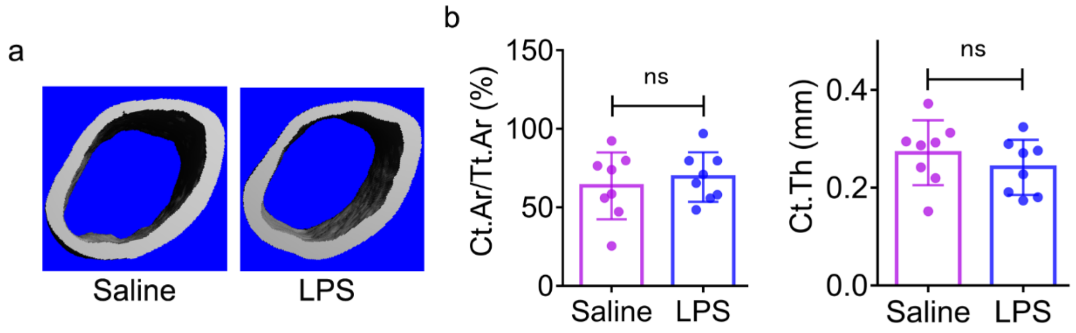

Figure S1

Supplementary Fig. 1: Cortical bone of femur in acute lung injury (ALI) of mice. (a) Representative 3D reconstruction images of femur mid-shaft cortical bone of ALI mice. **(b)** Cortical bone area fraction (Ct.Ar/Tt.Ar) and cortical bone thickness (Ct.Th) of femur mid-shaft of ALI mice determined by micro-CT (n=8). Data are representative of three independent experiments. Data were shown as the means ± s.d. ns: no significance.

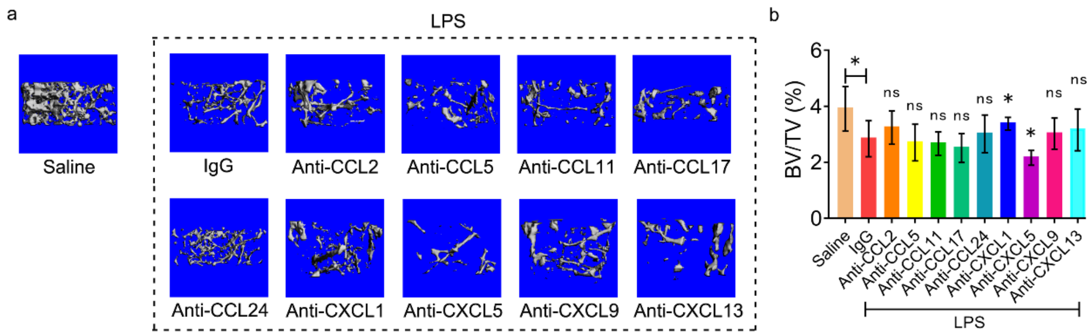

Figure S2

Supplementary Fig. 2: Trabecular bone mass of femur in ALI mice in response to administration of neutralization antibody against chemokines family members. (a) Representative 3D reconstruction images of distal femur trabecular bone of ALI mice in response to administration of neutralization antibody against CCL2, CCL5, CCL11, CCL17, CCL24, CXCL1, CXCL5, CXCL9, and CXCL13. **(b)** Trabecular bone volume fraction (BV/TV) of distal femur of ALI mice in response to administration of neutralization antibody against chemokines family members determined by micro-CT (n=8). Isotype IgG was used as negative control for neutralization antibody. Data are representative of three independent experiments. Data were shown as the means ± s.d. *: p<0.05, ns: no significance.

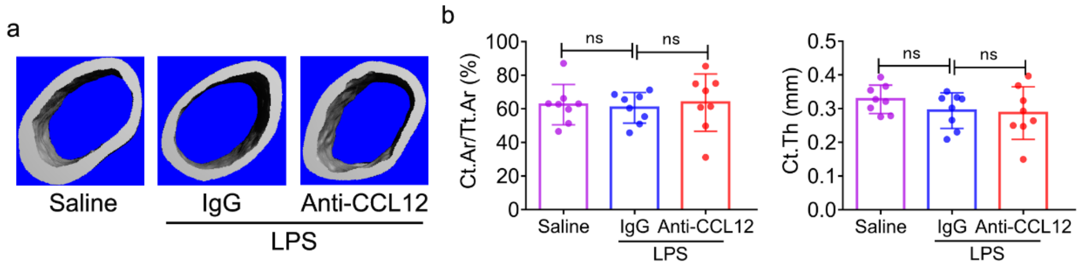

Figure S3

Supplementary Fig. 3: Cortical bone of femur in ALI mice in response to administration of neutralization antibody against CCL12. (a) Representative 3D reconstruction images of femur mid-shaft cortical bone of ALI mice in response to administration of neutralization antibody against CCL2. **(b)** Ct.Ar/Tt.Ar and Ct.Th of femur mid-shaft of ALI mice in response to administration of neutralization antibody against CCL12 determined by micro-CT (n=8). Isotype IgG was used as negative control for neutralization antibody. Data are representative of three independent experiments. Data were shown as the means ± s.d. ns: no significance.

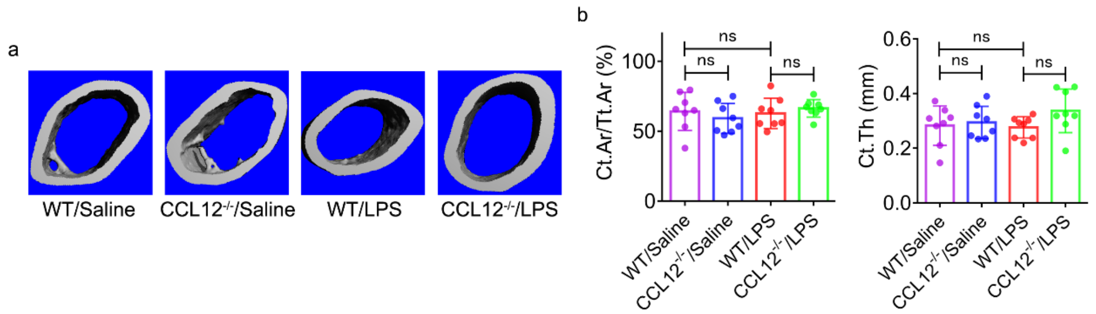

Figure S4

Supplementary Fig. 4: Cortical bone of femur in ALI CCL12$^{-/-}$ mice. (a) Representative 3D reconstruction images of femur mid-shaft cortical bone of ALI mice with global deletion of CCL12. **(b)** Ct.Ar/Tt.Ar and Ct.Th of femur mid-shaft of ALI mice with global deletion of CCL12 determined by micro-CT (n=8). Data are representative of three independent experiments. Data were shown as the means ± s.d. ns: no significance.

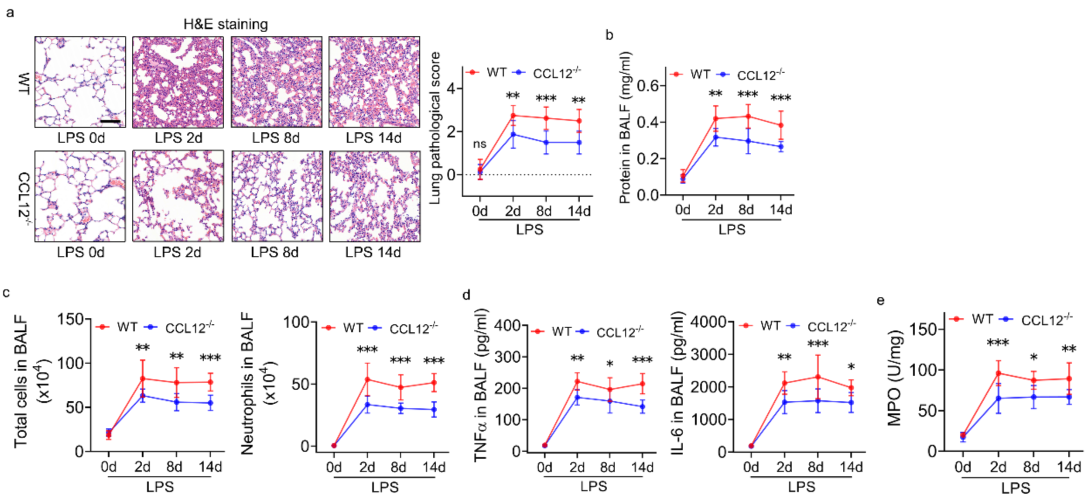

Figure S5

Supplementary Fig. 5: Acute lung injury at day 2,8, and 14 after LPS inhalation in CCL12$^{-/-}$ mice. (a) Lung histology by H&E staining and quantitative analysis of acute lung injury (ALI) in WT and CCL12$^{-/-}$ mice (n=8). Scale bar: 50 μm. **(b)** Total protein concentration in BALF of ALI mice with global deletion of CCL12 determined by BCA method (n=8). **(c)** Total cell and neutrophils counts in BALF of ALI mice with global deletion of CCL12 (n=8). **(d)** TNFα and IL-6 levels in BALF of ALI mice with global deletion of CCL12 determined by ELISA (n=8). **(e)** Myeloperoxidase (MPO) activity in lung tissues of ALI mice with global deletion of CCL12 (n=8). Data are representative of three independent experiments. Data were shown as the means ± s.d. *: p<0.05, **: p<0.01, ***: p<0.001, ns: no significance, WT *vs.* CCL12$^{-/-}$.

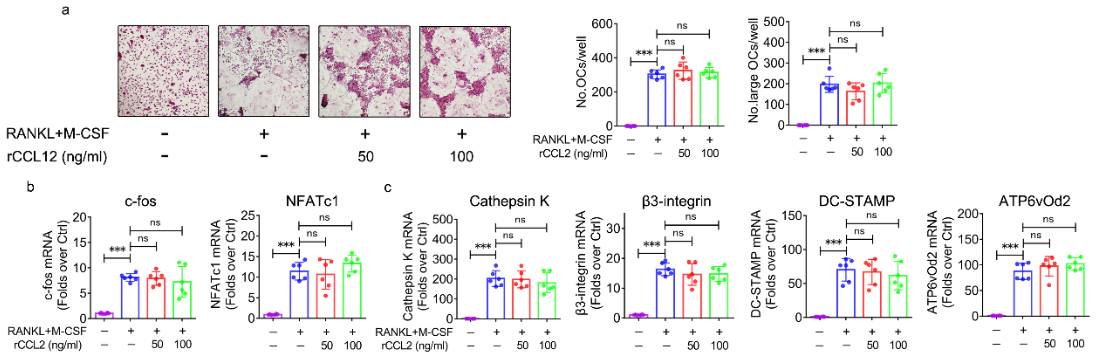

Figure S6

Supplementary Fig. 6: *In vitro* osteoclast differentiation of bone marrow monocytes in response to CCL12 treatment. (a) Trap staining images and quantification of *in vitro* BMMs culture in response to M-CSF/RANKL and recombinant CCL12 (50 and 100 ng/ml) treatment. **(b)** mRNA expression of key transcription factors of osteoclast differentiation including c-fos and NFATc1 in BMMs culture in response to M-CSF/RANKL and recombinant CCL12 (50 and 100 ng/ml) treatment determined by RT-qPCR (n=8). **(c)** mRNA expression of osteoclast differentiation markers including Cathepsin K, β3-integrin, DC-STAMP, and ATP6vOd2 in BMMs culture in response to M-CSF/RANKL and recombinant CCL12 (50 and 100 ng/ml) treatment determined by RT-qPCR (n=8). α-tubulin was used as internal control for qPCR. Data are representative of three independent experiments. Data were shown as the means ± s.d. ***: p<0.001, ns: no significance.

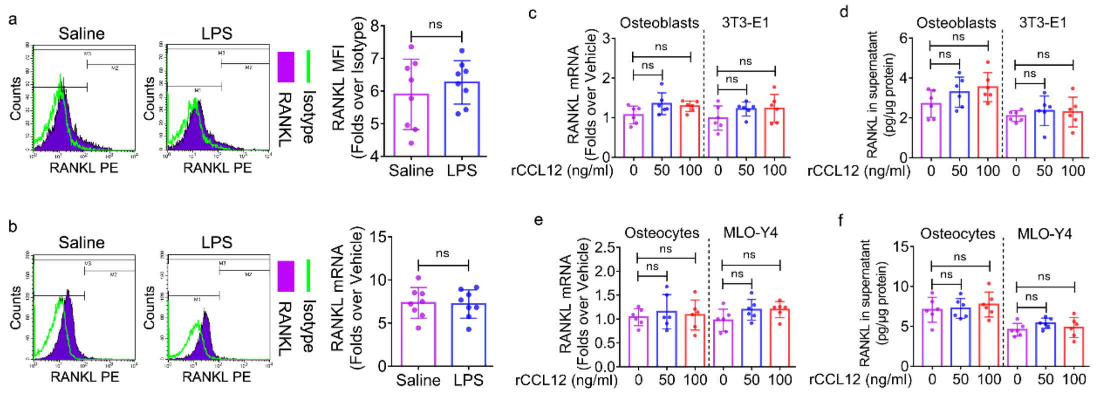

Figure S7

Supplementary Fig. 7: RANKL expression in osteoblasts and osteocytes of ALI mice and the effect of recombinant CCL12 on RANKL expression in *in vitro* osteoblasts and osteocytes cultures. (a) RANKL protein expression in osteoblasts from ALI mice determined by flow cytometry (n=8). **(b)** RANKL protein expression in osteocytes from ALI mice determined by flow cytometry (n=8). **(c)** RANKL mRNA expression in *in vitro* primary osteoblasts and 3T3-E1 cell line culture in response to 0, 50, and 100 ng/ml recombinant CCL12 treatment for 48 hours determined by RT-qPCR (n=8). **(d)** RANKL secretion level in the medium of *in vitro* primary osteoblasts and 3T3-E1 cell line culture in response to 0, 50, and 100 ng/ml recombinant CCL12 treatment for 48 hours determined by ELISA (n=8). **(e)** RANKL mRNA expression in *in vitro* primary osteocytes and MLO-Y4 cell line culture in response to 0, 50, and 100 ng/ml recombinant CCL12 treatment for 48 hours determined by RT-qPCR (n=8). **(f)** RANKL secretion level in the medium of *in vitro* primary osteocytes and MLO-Y4 cell line culture in response to 0, 50, and 100 ng/ml recombinant CCL12 treatment for 48 hours determined by ELISA (n=8). α-tubulin was used as internal control for qPCR. Data are representative of three independent experiments. Data were shown as the means ± s.d. ns: no significance.

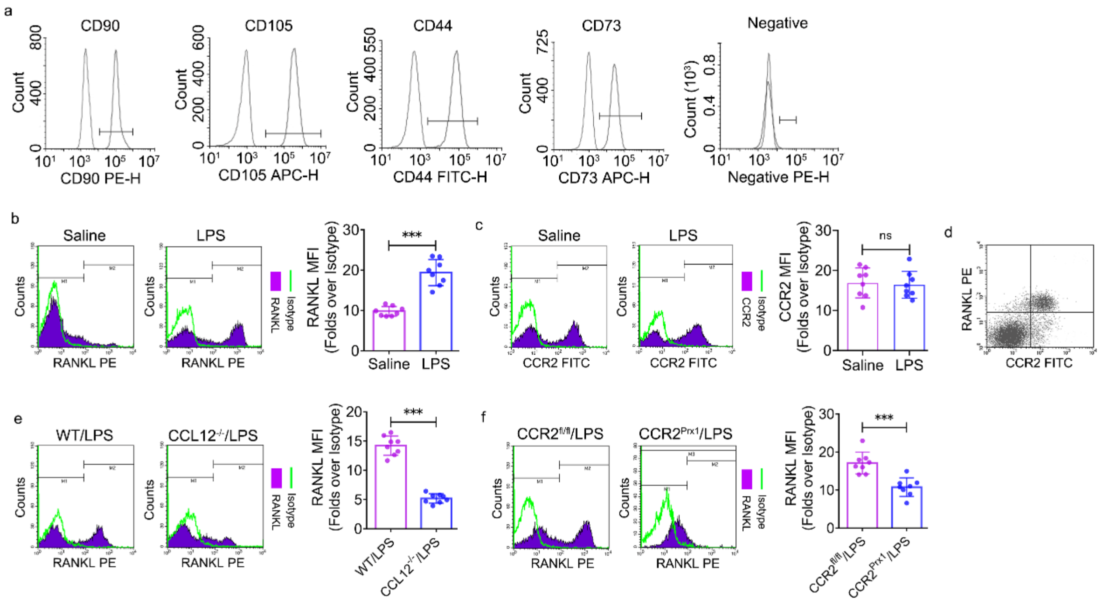

Figure S8

Supplementary Fig. 8: CCL12 increases RANKL production of BMSCs via CCR2 in ALI mice. (a) Flow cytometry analysis on mesenchymal lineage surface markers of BMSCs including $CD44^+$, $CD73^+$, $CD90^+$, $CD105^+$, $CD11b^-$, $CD19^-$, $CD34^-$, $CD45^-$, and $HLA\text{-}DR^-$. **(b)** RANKL protein expression in BMSCs from ALI mice determined by flow cytometry (n=8). **(c)** CCR2 protein expression in BMSCs from ALI mice determined by flow cytometry (n=8). **(d)** $RANKL^+CCR2^+$ BMSCs sorting from bone marrow of mice determined by flow cytometry. **(e)** RANKL protein expression in BMSCs from ALI mice with global deletion of CCL12 determined by flow cytometry (n=8). **(f)** RANKL protein expression in BMSCs with CCR2 deletion from ALI mice determined by flow cytometry (n=8). ***: p<0.001, ns: no significance.

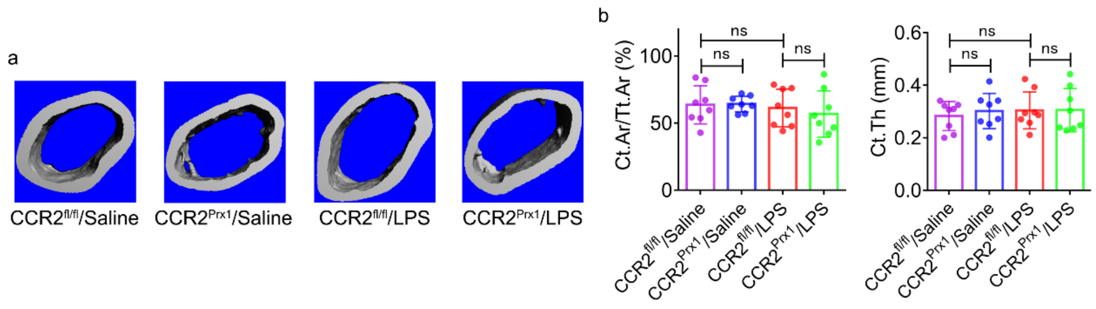

Figure S9

Supplementary Fig. 9: Cortical bone of femur in ALI mice with conditional deletion of CCR2 in BMSCs. (a) Representative 3D reconstruction images of femur mid-shaft cortical bone of ALI mice with conditional deletion of CCR2 in BMSCs. **(b)** Ct.Ar/Tt.Ar and Ct.Th of femur mid-shaft of ALI mice with conditional deletion of CCR2 in BMSCs determined by micro-CT (n=8). Data are representative of three independent experiments. Data were shown as the means ± s.d. ns: no significance.

RANKL promoter sequence

CACAGGCAGAGGAGGCAGAGATGGCAGAGAGGCACAGGCAGAGGCGA
GTGGATCTCTGAGTTTGAGGTCAGCCTGGTTCATATAGCAAGCTCCAGGC
CAGCCTAGTTTACATACAGAGAACTTGTCTCAAACAAATAAACCAAAACGA
CAACAAAACAATAACAAAAATATTTGTGCGCGCGCGTGTGCGTGCTTG
CGCACATGCCGGAGGAAAAGGATGAGGATTCTTCTGGTCAAATTCAGGT
CCTCAAGATAAGTTGCAAGTGCCTTCACCAGCTGAGCCGTCTTATTGGCC
CTCAGCATAAGGCTTTCTTAAGCAGTGAGGCTTAACTCTGACATTAGCTCT
GCTAACAGCTCTTTCCTGACTGTTCCATCATCCCCACGCTGGGAAGCCTG
CATGCAGTTTTGTACCCTCTACCCATCCCTTTGTCTAGTTCTTCCTGGCTC
ACCCACCCCATTCTTTTCCTACTTCATTCCTCTCTTACGGAGTCTATAGG
CTCATTGGAGCATCTGCCCCATTTGAAGTCACTTTTGAGATGAAGATAAAG
GCACTTGGGAGGGAGTTCTAGAATTTCCCCAAGTCTTCCCAATAGCCCGT
GAGGCTATATTTGGAGGGATAACTGAGGCTAAACCTCACGATTCTTGATG
GTGGTCTCCTCTAAGATTTTGAGAGTGGTGTACAGGAAAGGGCTTCGGA
CGGAGTTCACTAGAGCTGACTTTTTAAATCTTACAGAGGAAACTGAGGCC
CAGAGATGCAAAGGATAGGGGCCAGCCTAGAGAGCCAGAAACCAACCAC
TGGACCCAACCCACAGCCTCCACCTCAGAGGGCCCTGATGGGGAGGGA
GGAGGCGGGTCCCTGAGAGCGAAGAAGAGTGGGAGGGCGAAGGAAAG
GAAGGAGGGCAGATGTGGGAGTGAAAGAGGCACCCTCCTGGAGGCTGA
TTGGCTCTGGAGGCCAGCTCTCTCCACGAGGTTTATAAGAGTTAGGGCT
GCCTGGGGTACCCTGCCATCTCTCCCACGTCCCGGGGAGCCACTGCCA
GGACCTCTGTGAACCGGTCGGGGCGGGGGCCGCCTGGCCGGGAGTCT
GCTCGGCGGTGGGTGGCCGAGGAAGGGAGAGAACGATCGCGGAGCAG
GGCGCCCGAACTCCGGGCGCCGCGCCAT

Transcription start site ChIP region Putative STAT3/4 binding site

Putative STAT3 binding site

Supplementary Fig. 10

Supplementary Fig. 10: RANKL promoter sequence investigated in the current study. The DNA sequence of -1005bp~+156bp around RANKL transcription start site was shown. Transcription start site was labeled in red, putative STAT3 binding site was labeled in yellow, and STAT3/4 binding site was labeled in dark green, and ChIP region was labeled in blue.
